# Supplementary material for: Patterns of Intron Gain and Loss in Fungi
Source: PLoS Biol. 2004 Nov 30;2(12):e422. doi: 10.1371/journal.pbio.0020422 (PMC532390; doi:10.1371/journal.pbio.0020422)
Supplement: Table S1 — Also available at http://genes.mit.edu/NielsenEtAl/. (4.3 MB ZIP). [file pbio.0020422.st001.zip › NielsenEtAl/html/17.html]

AN0646.1.NCU04242.1.MG00976.1.FG09930.1


```
 CLUSTAL W (1.82) Multiple Sequence Alignments - Introns Inserted


Sequence 1: NCU04242.1	1093 aa
Sequence 2: FG09930.1	1083 aa
Sequence 3: MG00976.1	1105 aa
Sequence 4: AN0646.1	1062 aa
Alignment Length: 1131 aa
Number Identitical Residues: 688 aa
Alignment Score (without introns) 30984


MG00976.1 	---MEGAFTHLGNHLISDSAAAIKAGDTDDLSNIDADENLLYGKYGGRGGLGGARRRAAD
NCU04242.1	MANMEVSFTHLGNHLVSDSAAAIKAGGSDELSNIDPDENLLYGVYGGRGPRGNGRRRHDD
FG09930.1 	---MEGAFTHVGNHLISDSAAAIKAG-ADDLSTIDPDESLLYAKYGDR----NGRRRADD
AN0646.1  	----------------------------------------MDRTVGGP-----RRRRHDD
          	                                        :    *.       ***  *

MG00976.1 	DD-ETEALDDDDDESLASVPVDGMRGLSLKAAEEEKELPEHACA2YCGIHSPACVVKCLG
NCU04242.1	DDNETEVLDDDDDESLASVPVDGMKSLKLDAPVEEKELPPHACA~YCGIHSPSSVVKCLT
FG09930.1 	DDNQTEVFEEDDNDSLNSVPVDGLNGLKLKGKEEEKELPAHACA2YCGIHSPGCVVKCLT
AN0646.1  	DDEGPDNSDDDDLESTTSAPAN--KAKYPGELEEEKELAPHACA2YCGIHNPSSVVKCLA
          	**. .:  ::** :*  *.*.:  ..       *****. **** *****.*..***** 

MG00976.1 	CKKWFCNSRGNTSSSHIINHLVRARHKEVQLHPESTLGDTILECYNCGVRNVFLLGFIPA
NCU04242.1	CNKWFCSAKGSAFSSHIVNHLVRARHKEVQLHPESSLGDTVLECYNCGTKNVFILGFIPA
FG09930.1 	CNKWFCSARGNGTSTHIVNHLVRARHKEVQLHPESTLGDTVLECYNCGTKNAFLLGFIPA
AN0646.1  	CSKWFCSARGNTSSSHIVNHLVRARHKEVQLHPESSLGDTVLECYNCGTKNVFLLGFIPA
          	*.****.::*.  *:**:*****************:****:*******.:*.*:******

MG00976.1 	KSDTVVVLLCRQPCAANPSSKDMSWDISRWSPLLDERNFLSWLVAVPTDAEQLRARHLTP
NCU04242.1	KSDTVVVLLCRQPCGASTSTKDMSWDISRWQPLIEDRAFLNWLVTPPTDAEQLRARHLTP
FG09930.1 	KSDTVVVLLCRQPCAANTSNKDMNWDTSRWQPLIEERAFLPWLVATPSDSEQLRARHLTP
AN0646.1  	KSDTVVVLLCRQPCAAMPSSKDMNWDTSRWQPLIEDRSFLTWLVAAPSDQEQLRARHLSP
          	**************.* .*.***.** ***.**:::* ** ***: *:* ********:*

MG00976.1 	PMIAKLEELWKEDANATVDDLEKNEQIDDEPHPVLLKYDDPYHYQNIFGPLVKMESDYDK
NCU04242.1	PMIAKLEEMWKEAPNATVADLEKTAGVDDDPHPVLLKYDDPYHYQNIFGPLVKMESDYDK
FG09930.1 	NMIAKLEEMWKIEPKATVVDLEKAASIDDDPQPVLLNYDDPYHYQNIFGPLVKMESDYDK
AN0646.1  	QMIAKLEEMWKENSQATIADLEKATAVDDEPAPVLLRYDDAFQYQNIFGPLVKIEADYDR
          	 *******:**  .:**: ****   :**:* ****.***.::**********:*:***:

MG00976.1 	KLKEAQSEDGLVVRWDYGLNNKHLASFILPKIESGDVKLAVGDEMRLKYKGELRPQWEGV
NCU04242.1	KLKEAQSEDGLQVRWHLGLNSKHVASFILPKIESGDVKLAVGDEMRLKYKGELRPPWEGV
FG09930.1 	KLKEAQSEDGLLVRWDYGLNNKHLVSFNLHKIESGDVKLAVGDEMRLRYKGELRSPWEGV
AN0646.1  	KLKESQSQDGLIVRWDLGLNNKHLASFILPKLELGDVKLAVGDEMRLKYTGELRPKWEGV
          	****:**:*** ***. ***.**:.** * *:* *************:*.****. ****

MG00976.1 	GYVVKIPNNQSDEVTLELRKSGNDKSVPTECTHNFSADYVWKATSYDRMQLAMRNFAVDE
NCU04242.1	GYVIKIPNNQSDEVEVELRKSANDKSVPTECTHNFSADYVWKATSYDRMQLAMKTFAVDE
FG09930.1 	GYVIKIPNNQSDEVTLELRKTGNEKLVPTDLSHNFSADYVWKATSYDRMQLAMKTFAVDD
AN0646.1  	GYVIKIPNNQSDEVTIELRAKGDHKSVPTECTHNFTADYVWKSTSFDRMQLAMKTFAVDE
          	***:********** :*** ..:.* ***: :***:******:**:*******:.****:

MG00976.1 	MSVSGYIFHKLLGHEVAVAPMRTTMPKKFTAPGLPDLNGSQISAIKAVLSTPLSLIQGPP
NCU04242.1	MSVSGYIFHKLLGHEVQVAPTKITMPKKFHVPGLPELNASQIAAIKQVLSNPLSLIQGPP
FG09930.1 	MSVSGYIFHTLLGHEVQLQVMKTNLPKKWSAPGLPDLNPSQVGAIKAVLQKPLSLIQGPP
AN0646.1  	MSVSGYIFHRLLGHEVAAAPMKTQMPKKFSVPGLPELNGSQINAVKSVLQRPLSLIQGPP
          	********* ******     :  :***: .****:** **: *:* **. *********

MG00976.1 	GTGKTVTSATIIYHLAKMNGSQVLVCAPSNVAVDQLCERVHRTGLKVVRLTAKSREDVES
NCU04242.1	GTGKTVTSATIIYHLAKMSNSQVLVCAPSNVAVDQLCERIHRTGLKVVRLTAKSREDVES
FG09930.1 	GTGKTVTSATIIYHLAKMSGNQVLVCAPSNVAVDQLCERVHRTGLKVVRLTAKSREDVES
AN0646.1  	GTGKTVTSATIIYHLAKLNGGQVLVCAPSNVAVDQLCERIHRTGLKTVRVTAKSREDVES
          	*****************:...******************:******.**:**********

MG00976.1 	SVSFLALHEQVRMNDTNPELAKLQQLKSELGELSSQDEKKFKQLTKAAEREILSNADVVC
NCU04242.1	SVSFLALHEQVRMNTTNKELDGLVKLKTETGELSSQDEKRFKQLTRQAEREILQNADVVC
FG09930.1 	SVSFLALHEQVRMSEHNSELVKLSQLKNELGELSSQDEKKYKQLTKIAERDILNNADVVC
AN0646.1  	PVGFLSLHEQVRLNDSNIELIKLNQLKSELGELSSQDEKRLKQLTRAAEREILNNADVIC
          	.*.**:******:.  * **  * :**.* *********: ****: ***:**.****:*

MG00976.1 	CTCVGAGDPRLAKLKFRNVLIDESTQSAEPECMIPLVLGCKQVVLVGDHKQLGPVIMNKK
NCU04242.1	CTCVGAGDPRLSKMKFRNVLIDESTQSAEPECMIPLVLGCKQVVLVGDHKQLGPVIMNKK
FG09930.1 	CTCVGAGDPRLSKMKFRNVLIDESTQSAEPECMIPLVLGCKQVVLVGDHKQLGPVIMNKK
AN0646.1  	CTCVGAGDPRLAKLKFRTVLIDESTQSAEPECMIPLVLGCKQVVLVGDHQQLGPVIMNKK
          	***********:*:***.*******************************:**********

MG00976.1 	AAKAGLNQSLFERLVKLGLTPIRLNVQYRMHPCLSEFPSNMFYEGSLQNGVTIQQRLRRD
NCU04242.1	AAKAGLNQSLFERLVKLQFTPIRLKVQYRMHPCLSEFPSNMFYEGSLQNGVTAAERLRKD
FG09930.1 	AAKAGLNQSLFERLVNLKLSPIRLNIQYRMHPCLSEFPSNMFYDGSLQNGVTHENRLRKD
AN0646.1  	AAKAGLNQSLFERLVILGCSPIRLNVQYRMHPCLSEFPSNMFYEGSLQNGITSFDRLRRE
          	*************** *  :****::*****************:******:*  :***::

MG00976.1 	VDFPWPVADMPMMFWSNLGNEEISASGTSYLNRTEASNVEKLVTRFFKAGVKPGDIGVVT
NCU04242.1	VDFPWPVPETPMMFWSNLGNEEISASGTSYLNRTEAANVEKIVTRFFKAGVKPADIGVIT
FG09930.1 	VDFPWPVGEMPMMFWSNLGHEEISASGTSYLNRTEASNVEKAVTRFFKAGVKPADIGVIT
AN0646.1  	VDFPWPILDSPMMFWSNLGNEEISASGTSYLNRTEAANVEKIVTRFFKAGVQPKDIGIIT
          	******: : *********:****************:**** *********:* ***::*

MG00976.1 	PYEGQRSYIVSTMQNTGTFKKESYKEVEVASVDAFQGREKDFIVLSCVRSNESQGIGFLS
NCU04242.1	PYEGQRSYIVNTMQNTGTFKKESYREVEVASVDAFQGREKDFIVLSCVRSNENQGIGFLS
FG09930.1 	PYEGQRSYIVTTMQNSGTYKKEYYKEVEVASVDAFQGREKDFIVLSCVRSNDNQGIGFLS
AN0646.1  	PYEGQRSYIVSSMQATGTFKKEHYKEIEVASVDAFQGREKDFIILSCVRSNDHQGIGFLS
          	**********.:** :**:*** *:*:****************:*******: *******

MG00976.1 	DPRRLNVALTRAKYGVVIIGNPKVLSKHELWHHLLIHFRDRKCLVDGPLTNLQASLLQFS
NCU04242.1	DPRRLNVALTRAKYGLVIIGNPKVLCKHELWHHLLVHFKDKKCLVEGPLTNLQPSLLQFG
FG09930.1 	DPRRLNVALTRAKYGLVILGNPKVLSKHELWHNLLVHFKDRKCFVEGPLTNLQACLLQFS
AN0646.1  	DPRRLNVALTRAKYGLVILGNPKVLSKHPLWNCLLQHFKERHTLVEGPLSNLQESLIQFS
          	***************:**:******.** **: ** **:::: :*:***:*** .*:**.

MG00976.1 	KPKTPFRPARFSQQHSQYAPSQAGYANGRFPGSNLNGGSGRDFDTGSMMSYIPDDVSSLH
NCU04242.1	RPRQAYRPQ---RSHTQHVAS--GPSNG-RFAAPLTAGSVRDYETGSMVSYIPDDVSSIH
FG09930.1 	RPKVSFRQK---NQQPQFGAG--SYSNGGRFNPPPSG---RDFDLGSMVSYIPDDVSSVH
AN0646.1  	RPKQAYRGP----QRFQMAYNHVSNVTSGFMNG-RNGHRNEFHDTGSVVGYIPDDVSSVH
          	:*: .:*      .: *   .  .  ..       ..   . .: **::.********:*

MG00976.1 	SSSLGGAGLASAYPAMFSNFNPEQWPGLPGVPGHGRSGNRNRGRGAESIAGESVANSEYT
NCU04242.1	SSALGGAALSSGYPAMFSNFHPEAWAGLPCAGPNGRPGAKGRGRATESIAGESVANSEFT
FG09930.1 	GSAFGGASLNSAFPPMFSSFTPDQWPGLPGVAAPGR-GGKSRGRATESIAGESVANSEYT
AN0646.1  	SSALGGVGIPSGYPPMFQQFA-DSWPSLPGA----RRANGNRGKGAPSIAGDSVAATES-
          	.*::**..: *.:*.**..*  : *..** .    * .  .**:.: ****:*** :*  

MG00976.1 	DASSSVIGGKGIGQGGVSLGAGIGAGIGAGLHDAVGAGRPTSYSHSDRLRQYVESGG---
NCU04242.1	DATSSVIGGKGIGQGGASLGA--------GLSEAIGSARPTSYSQSDRLKQYVESNG---
FG09930.1 	DASSSVIGGKGVGQGGVSLGA--------GLHDAVTGMRPVSYSQSDRLKQYVESSG---
AN0646.1  	DITGSIIDGKGPEQGGVSLAG----------LSINDMTKQPSLSQSDRLKRYVESGARDP
          	* :.*:*.***  ***.**..           .     :  * *:****::****.. ..

MG00976.1 	-------------------RMTGPNNGYRRYDDDQQSISTAFASQAGGTGYD
NCU04242.1	-------------------RMGVGNGY-RRYDDDEKSVSTAFASQIG-TGFD
FG09930.1 	-------------------RMVPGSGYGRRFDDDEKSVSTAFHSQIG-GGYD
AN0646.1  	YKPGAPDSGSIFGGSSASIRVTRGIPGHIHDDDDARSVSTAFASQVG-GNYD
          	 ...:..:.:  ..:::: *:        : *** :*:**** ** *  .:*
```
